# An automated system for liquid-liquid extraction in monosegmented flow analysis

**DOI:** 10.1155/S1463924697000059

**Published:** 1997

**Authors:** Ileana Facchin, Jarbas J. R. Rohwedder, Celio Pasquini

**Affiliations:** Instituto de Química Universidade Estadual de Campinas Caixa Postal 6154 Campinas SP CEP 13083-970 Brazil

## Abstract

An automated system to perform liquid-liquid extraction in monosegmented flow analysis is described. The system is controlled by a microcomputer that can track the localization of the aqueous monosegmented sample in the manifold. Optical switches are employed to sense the gas-liquid interface of the air bubbles that define the monosegment. The logical level changes, generated by the switches, are flagged by the computer through a home-made interface that also contains the analogue-to-digital converter for signal acquisition. The sequence of operations, necessary for a single extraction or for concentration of the analyte in the organic phase, is triggered by these logical transitions. The system was evaluated for extraction of Cd(II), Cu(II) and Zn(II) and concentration of Cd(II) from aqueous solutions at pH 9.9 (NH_3_/NH_4_Cl buffer) into chloroform containing PAN (1-(2-pyridylazo)-2-naphthol) . The results show a mean repeatability of 3% (rsd) for a 2.0 mg l^-1^ Cd(II) solution and a linear increase of the concentration factor for a 0.5mg l^-1^ Cd(II) solution observed for up to nine extraction cycles.


Journal of Automatic Chemistry, Vol. 19, No. 2 (March–April 1997) pp. 33–38

### An automated system for liquid–liquid extraction in monosegmented flow analysis

**Ileana Facchin, Jarbas J. R. Rohwedder* and Celio Pasquini**

*Instituto de Química, Universidade Estadual de Campinas, Caixa Postal 6154, CEP 13083-970, Campinas, SP, Brazil*

*An automated system to perform liquid–liquid extraction in monosegmented flow analysis is described. The system is controlled by a microcomputer that can track the localization of the aqueous monosegmented sample in the manifold. Optical switches are employed to sense the gas–liquid interface of the air bubbles that define the monosegment. The logical level changes, generated by the switches, are flagged by the computer through a home-made interface that also contains the analogue-to-digital converter for signal acquisition. The sequence of operations, necessary for a single extraction or for concentration of the analyte in the organic phase, is triggered by these logical transitions. The system was evaluated for extraction of Cd(II), Cu(II) and Zn(II) and concentration of Cd(II) from aqueous solutions at pH 9·9 ($NH_3/NH_4Cl$ buffer) into chloroform containing PAN (1-(2-pyridylazo)-2-naphthol). The results show a mean repeatability of 3% (rsd) for a 2·0 mg $l^{-1}$ Cd(II) solution and a linear increase of the concentration factor for a 0·5 mg $l^{-1}$ Cd(II) solution observed for up to nine extraction cycles.*

Since its introduction in 1978 [1–2], automated liquid–liquid extraction made by using flow systems (such as flow injection [FI]) has been used for a variety of analytical applications. Typically, the procedure for performing this automated technique involves the injection of an aqueous sample into an aqueous carrier stream, to which an organic phase is continuously added by confluence. The segmented stream obtained is pumped through a coil where extraction occurs. The two immiscible phases need to be separated before detection and the organic phase carried through a flow cell for measurement.

The segmentation and the phase-separation stages are frequently reported as being critical for achieving reproducible results [3]. To avoid problems in these stages, many alternative FIA manifolds have been developed. Most of the analysers (without phase separation) require computer control and allow monitoring of the analyte contained in the segments inside the extraction coil with 'on tube' detection [4–7]. Alternatively, iterative reversal of the flow direction is used, where the acceptor phase movement is controlled [8, 9]. Furthermore, extractive membranes [10–13], zone sampling [14] or trapped extractants [15] can be used.

Monosegmented Flow Analysis (MSFA) was recently evaluated for performing liquid–liquid extraction, by employing both two-phase [16] and single-phase [17] processes. The monosegmented technique employs two air bubbles that permit precise sample localization, allowing the detection to be easily made without phase separation. Furthermore, rigid control of segmentation is not necessary and the analyser construction is, in this way, simplified.

This paper describes an automated monosegmented flow analyser for extraction and concentration of metals, making use of a chloroform organic phase containing 1-(2-pyridylazo)-2-naphthol (PAN) as extracting agent. The system has been evaluated by using spectrophotometric detection of the metal-PAN complex extracted by the organic phase.

### Experimental

### *Reagents*

All chemicals were of analytical grade and the water used was distilled and deionized (Milli-Q Plus Ultra-Pure Water System). The 0·05% (w/v) extracting solution was prepared by dilution of a PAN reagent (Carlo Erba) in chloroform which contained amylene as preservative. Both the reagent and the solvent were used without previous treatment. Sample solutions of Cd(II), Cu(II) and Zn(II) were prepared by suitable dilution of stock solutions containing 100 mg $l^{-1}$ of the metal ion in $1{\cdot}0 \times 10^{-3}$ mol $l^{-1}$ nitric acid. The pH of the standard solutions was adjusted to 9·9 in line by merging them with the buffer solution before the injection port. The same buffer solution was employed as carrier solution and was prepared by dissolving 6·0 ml of aqueous 28–30% ammonia solution and 1·00 g of $NH_4Cl$ in 1 l of deionized water and adjusting the pH to 9·9.

### *Monosegmented flow manifold*

A schematic diagram of the monosegmented analyser for liquid–liquid extraction is shown in figure 1. An Ismatec MP-13R peristaltic pump was used. Viton and Tygon pump tubes were employed for organic and aqueous solutions, respectively. A 200 µl sample was introduced between two 50 µl air bubbles employing a sample introduction device [18]. The coils and loops were made of poly(tetrafluoroethylene) (PTFE), 0·5 mm i.d. The straight Pyrex glass extraction tube had an inner diameter of 2·0 mm and was 50 cm long. Attached to this tube, 45 cm from the point where the glass tube was connected to the sample introduction device, was the spectrophotometric detector, based on a green (560 nm) light-emitting diode (LED) and a light-dependent resistor (LDR). The resistance of the LDR was evaluated by a simple Wheatstone bridge circuit [19]. On-line detection occurs as a result of an imbalance in the potential of the bridge which was presented directly to

* Author to whom correspondence should be addressed.

0142-0453/97 $12·00 © 1997 Taylor & Francis Ltd

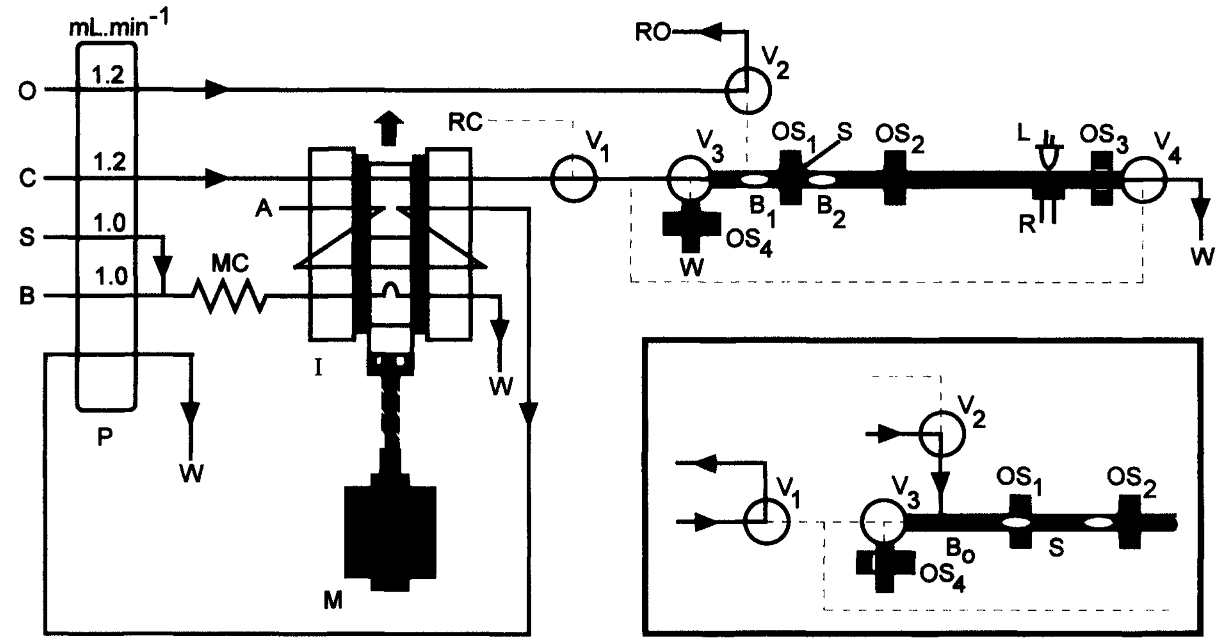

*Figure 1. Automatic monosegmented flow manifold for liquid–liquid extraction. P = peristaltic pump; S = sample; O = organic phase; C = carrier stream; B = buffer solution; MC = mixing coil; A = air; I = injection valve; M = stepper motor; W = waste; RC and RO denote, respectively, the return path for carrier and organic stream to reservoir flasks; L = light emitting diode (560 nm); R = light dependent resistor; $V_1$, $V_2$, $V_3$ and $V_4$ = three-way solenoid micro-valves; $OS_1$, $OS_2$, $OS_3$ and $OS_4$ = opto-switches; $B_1$ and $B_2$ = air bubbles; $B_o$ = organic segment. The glass extraction tube is shown between $V_3$ and $V_4$.*

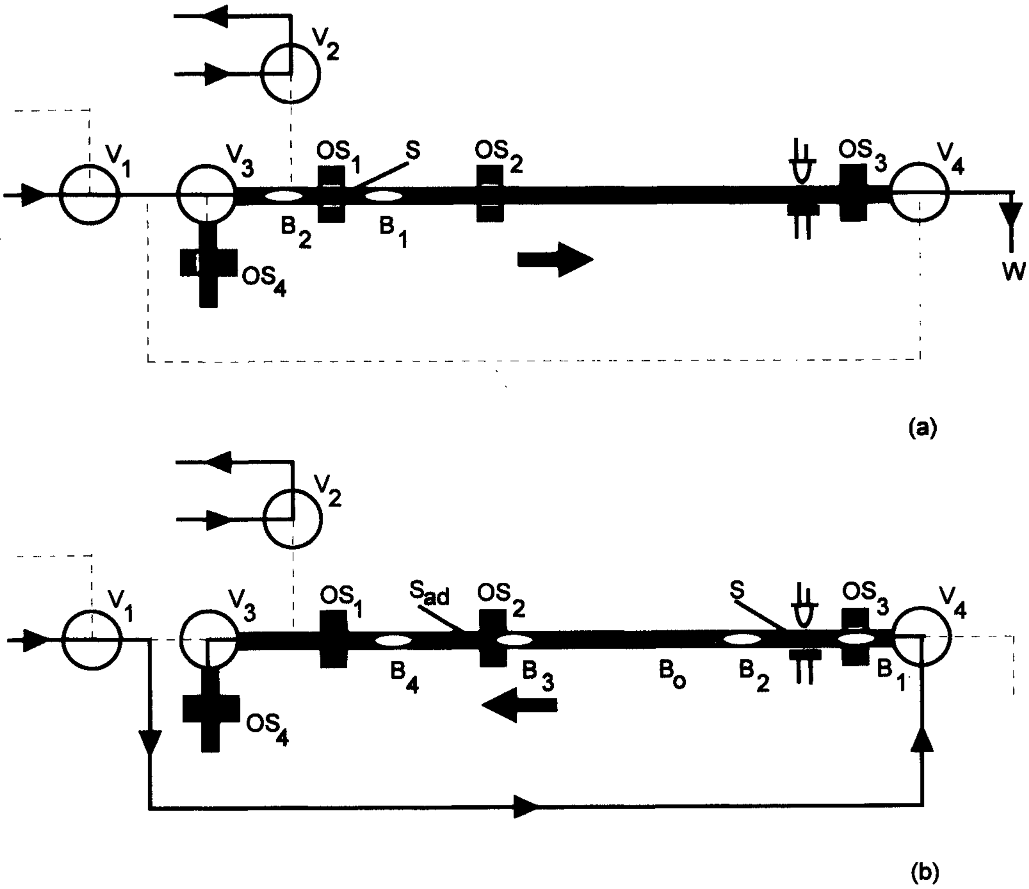

*Figure 2. Detail of the monosegmented flow analyser showing the phase arrangement pattern used to perform analyte concentration. $B_3$ and $B_4$ = air bubbles of the additional sample monosegment; $S_{ad}$ = additional sample segment. Other symbols as figure 1. a, system with the first sample monosegment; b, system in reverse flow to extract the analyte from the additional sample monosegment and to remove this aqueous segment through $V_3$.*

a home-made interface. Infrared opto-switches (PCTS-2103) ($OS_1$ to $OS_4$) and four three-way valves (N Research Model 161TO11; 12 V; 80 mA) were also used. The glass tube was rinsed, before use, with detergent, water and nitric acid (10% v/v), in order to remove contaminants. To avoid carry-over between samples, a 1·0 mol $l^{-1}$ nitric acid washing solution was introduced into the system in place of the sample.

### *Extraction procedures*

The automated analyser can perform two different procedures. In the first, a single aqueous sample monosegment is introduced in a buffered carrier stream followed by the extracting solution segment. The phase arrangement pattern, see figure 1, is transported, by the carrier fluid, through the glass extraction tube, towards the detector. Another way to process the extraction, involves concentration of the analyte and the introduction of additional sample monosegments after the organic phase, see figure 2.

### *The automated monosegmented flow analyser*

An IBM-PC 286 AT compatible microcomputer was used to control the flow system and for data acquisition. A home-made eight bit parallel asynchronous interface [20] was used for analogue-to-digital conversion, acting on electromechanical valves and acquiring the TTL logic state of the optical switches. The interface communicates with the microcomputer through a user port, which is based on a CI 8255 [20] and is plugged into an expansion slot inside the microcomputer. An eight bit analogue-to-digital converter (ADC 0808) was employed for data acquisition from the detector; this was based on a LDR.

The circuit diagram of the optical switches, used to monitor the passage of the bubbles in the glass tube, was described previously [21]. The TTL logical level is low when the glass tube is filled with an aqueous solution; the level changes to high when an air bubble passes these switches. A low TTL level was also observed for the organic phase.

### *Software to control the automated flow system*

The basic software to drive the user port and interface has been described previously [22, 23]. The program was written in Quick-Basic 4.5 and a flow chart is shown in figure 3. Cyclic operation of the system starts by turning the peristaltic pump on, the sample introduction device remains in the sampling position, and the electromechanical valves ($V_1$–$V_4$) are off. While in this position, the buffered carrier stream is continuously pumped towards the detector, and the organic phase returns to the flask reservoir (see figure 1). When the sample loop has been filled with sample solution, the stepper motor is turned on and the sample introduction device is placed in the injection position. The TTL logical level of the optical switch $OS_1$ is observed. After the passage of the air bubbles $B_1$ and $B_2$ through the optical switch $OS_1$, the electromechanical valves $V_1$ and $V_2$ are turned on. In this state, the organic phase is added to the carrier stream, for a time interval $t_0$, while the buffered carrier steam is returned to the flask reservoir (see figure 1). The sample introduction device is returned automatically to the sampling position by moving the stepper motor in the opposite direction. At this point, two different procedures can be followed: if the number of cycles ($N_C$) is equal to zero a single extraction is performed (procedure A), otherwise, ($N_C > 0$) the concentration of the analyte is performed (procedure B) through the injection of a user defined number of additional aqueous samples. In the first case, the TTL logical level of the optical switch $OS_3$ is monitored. After the passage of the air bubble 1 through this switch, the data acquisition is initiated and extends for a time interval $t_R$, long enough to allow all the organic phase to flow through the detector. Replicates or new sample extractions are performed following procedures C and E, respectively.

In the second case (procedure B, for analyte concentration), the TTL logical level of the optical switch $OS_2$ is evaluated. After the passage of the air bubbles $B_1$ and $B_2$ through this switch, an additional sample is injected (see figure 2[*a*]). The TTL logical level of the optical switch $OS_3$ is observed. When the bubble $B_1$ changes the TTL level of this switch, the electromechanical valves $V_3$ and $V_4$ are turned on. At this point, the flow stream is reversed as shown in figure 2[*b*]. Finally, the sample injection device is moved to the sampling position. Valves $V_3$ and $V_4$ are turned off when the air bubbles $B_3$ and $B_4$ pass through the optical switch $OS_4$. Additional sample is eliminated through $V_4$. A new cycle for concentrating the analyte is possible by following procedure D (injection of another aqueous sample). After this point, procedure A is followed and the enriched organic phase is monitored by the detector.

### Results and discussion

The analyte transference mechanism between two immiscible phases in monosegmented flow extraction is associated with an adsorption/desorption process on the glass wall of the extraction tube. Therefore the analyte, present in the aqueous phase, in a suitably complexed form, is adsorbed on the glass surface. When the organic phase, containing the complexing reagent (PAN) flows through the extraction tube, it removes the adsorbed analyte and, simultaneously, promotes the reaction for its spectrophotometric detection. In view of this mechanism, the use of additional sample injections after the organic phase were investigated to obtain the concentration of the analyte in the organic phase. In this way, while the organic phase removes the analyte of the first injected monosegment of the aqueous sample (S), the metallic specimens present in the additional monosegment ($S_{ad}$), carried down-stream by the buffered solution, are adsorbed on the glass surface tube. When the flow direction is changed (before the organic phase segment reaches the detector) (see figure 2), the extracting organic solution removes the adsorbed analyte left on the glass wall by the additional aqueous segment while it is pumped to the waste. Then a new sample monosegment can be injected, starting another cycle. The number of cycles performed determines the increase of signal

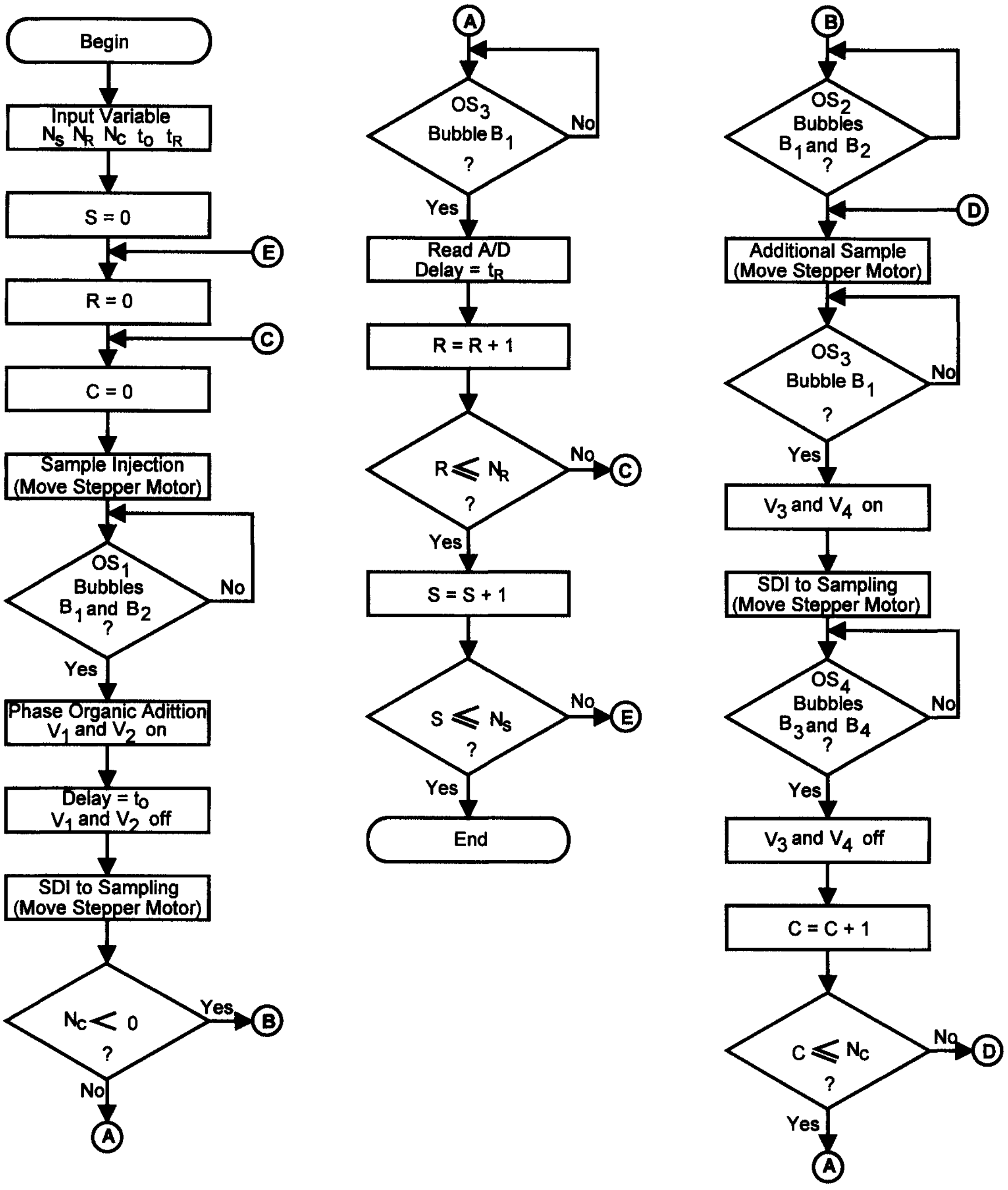

*Figure 3. Flow chart of the analyser controller program. See text for detailed description. A: single extraction; B: analyte concentration.*

intensity. Examples of signals obtained for 0·5 mg $l^{-1}$ Cd(II) extraction and concentration are shown in figure 4, where only the organic portion is represented along the base line, established while the buffer carrier solution is passing through the LED/LDR detection system.

Analytical curves for the extraction of Cd(II), Cu(II) and Zn(II) with PAN are shown in figure 5. The flow extraction procedure employed the same volume of sample and organic extractant (200 μl), without any additional monosegment sample ($S_{ad}$). The analytical parameter evaluated in this case was the peak area. However, signal height could also be used, leading to highly reproducible results. The curves are linear for all metal ions up to 2·5 mg $l^{-1}$. The analytical curve for Zn(II) shows a sigmoid format, with sensitivity decreasing at low concentrations [16]. Signals obtained for the three metals at 1 and 2 mg $l^{-1}$ show relative standard deviations lower than 3·0% for six measurements at each concentration.

The detection limit (DL), estimated as three times the signal-to-noise ratio [24], was equal to 50 and 60 μg $l^{-1}$ for Cd(II) and Cu(II), respectively. However, it was not possible to estimate the DL and Zn(II), because the signal intensity for this analyte is reduced for concentrations below 0·5 mg $l^{-1}$ of the metal ion.

The increase of signal intensities based on introduction of additional samples ($S_{ad}$) was investigated for a 0·5 mg $l^{-1}$ Cd(II) solution using up to nine concentration cycles. The graph obtained is shown in figure 6 and demonstrates

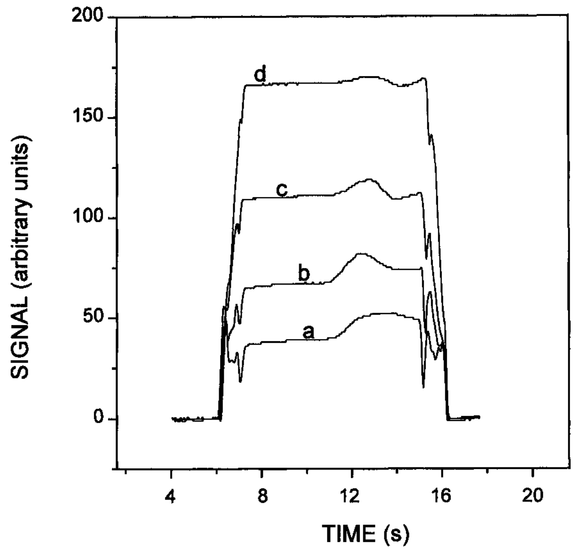

*Figure 4. Typical analytical signal (organic phase passage) obtained with procedure B for analyte concentration. The labelled portions refer to: a = blank; b, c and d = signals for 0·5 mg $l^{-1}$ Cd(II) employing one, four and nine cycles, respectively.*

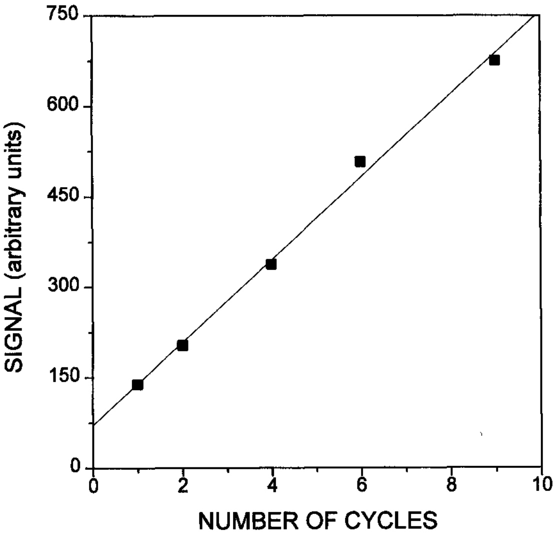

*Figure 6. Effect of the number of cycles on the area under the analytical signal for a 0·5 mg $l^{-1}$ Cd(II) solution. Sample and organic phase volume, 200 μl. Carrier buffer flow rate, 1·2 ml $min^{-1}$.*

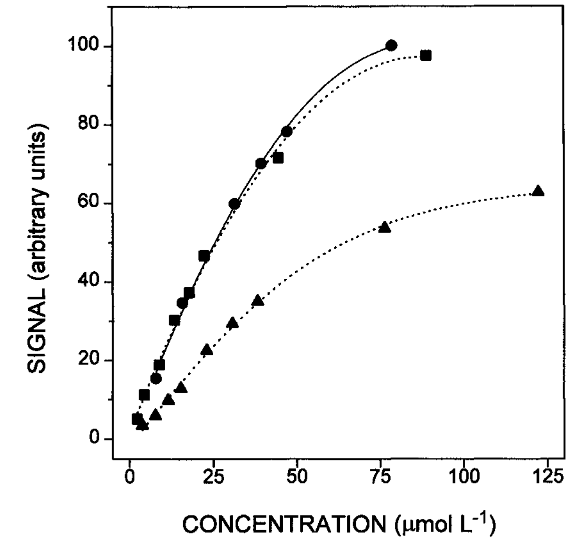

*Figure 5. Analytical curves obtained for single extractions of Cd(II) (■), Cu(II) (●) and Zn(II) (▲) in the $NH_3$-$NH_4Cl$ buffer at pH 9·9. Sample and organic phase volume, 200 μl. Carrier buffer flow rate, 1·2 ml $min^{-1}$.*

linear behaviour in the range evaluated. Furthermore, the integrated signal increases about 71% for each additional cycle. This result agrees with the mass balance results obtained for a single extraction of Cd(II) (procedure A), revealing that most of the metal fraction adsorbed is removed after a single pass of the organic phase segment through the glass tube.

### Conclusions

The use of a monosegmented flow system to perform liquid–liquid extraction was recently demonstrated [16]. In this work the importance of the two air bubbles for the analyser operation was described. These bubbles allow identification of the fraction of the flow that contains the sample, permitting organic phase addition in a reproducible way. Furthermore, the air bubbles allow the stream portion (containing the organic phase segment) that must be monitored in order to generate the analytical signal to be localized. In this way, a total recovery of the organic phase and the dispensing of the use of a separation device are achieved.

This paper describes the construction of an automated analyser which has a simple manifold and attained analyte concentration by reversing the carrier flow stream, thus introducing additional monosegmented aqueous samples. An increase in sensitivity was observed and the consumption of organic phase was reduced. This last feature is important in terms of cost and in the decrease of chemical waste production. Furthermore, as the glass surface has the adsorbed analyte removed each time organic phase is moved through the tube, no saturation state is obtained and the concentration procedure can be run as many times as desired. Limitations for this procedure can be, eventually, the long time interval for each determination necessary to achieve a very high factor of concentration, and/or the depletion of extracting reagent in the organic phase. Each concentration cycle in the present manifold takes 60 s to be processed.

Based on the adsorption/desorption mechanism to process liquid–liquid extraction by MSFA, additional investigation on the chemical modification of the glass tube surface would be of interest because it could improve the efficiency and/or selectivity, when interferents are present. Also, based on the proposed extraction mechanism, the use of masking agents and the development of coupled systems, that can transport the organic phase to a selective detector for final quantification or the aqueous phase to parallel flow manifold, will be evaluated in the future.

### Acknowledgements

The authors are grateful to C. H. Collins for manuscript revision. Ileana Facchin is grateful to CNPq for the PhD fellowship.

### References

1. Karlberg, B. and Thelander, S., *Analytica Chimica Acta*, **98** (1978), 1.
2. Bergamin, F°, H., Medeiros, J. X., Reis, B. F. and Zagatto, E. A. G., *Analytica Chimica Acta*, **101** (1978), 9.
3. Kuban, V., *CRC Critical Review Analytical Chemistry*, **22** (1991), 477.
4. Lucy, C. A. and Cantwell, F. F., *Analytical Chemistry*, **61** (1989), 107.
5. Thommen, C., Fromageat, A., Obergfell, P. and Widmer, H. M., *Analytica Chimica Acta*, **234** (1990), 141.
6. Liu, H. G. and Dasgupta, P. K., *Analytica Chimica Acta*, **288** (1994), 237.
7. Hortiz-Boyer, F., García-Mesa, J. A. and Luque de Castro, M. D., *Analytical Chemistry*, **66** (1994), 2794.
8. Canete, F., Rios, A., Luque de Castro, M. D. and Valcárcel, M., *Analytical Chemistry*, **60** (1988), 2354.
9. García-Mesa, J. A., Linares, P., Luque de Castro, M. D. and Valcárcel, M., *Analytica Chimica Acta*, **235** (1990), 441.
10. Moskvin, L. N. and Simon, J., *Talanta*, **41** (1994), 1765.
11. Barnes, D. E. and van Staden, J. F., *Analytica Chimica Acta*, **261** (1992), 441.
12. Rodriguez-Gonzalo, E., Perez-Pavon, J. L., Ruzicka, J., Christian, G. D. and Olson, D. C., *Analytica Chimica Acta*, **259** (1992), 37.
13. Melcher, R. G., *Analytica Chimica Acta*, **214** (1988), 299.
14. Lindgren, C. C. and Dasgupta, P. K., *Talanta*, **39** (1992), 101.
15. Agudo, M., Ríos, A. and Valcárcel, M., *Analytical Chemistry*, **65** (1993), 2941.
16. Facchin, I. and Pasquini, C., *Analytica Chimica Acta*, **308** (1995), 231.
17. Facchin, I., Martins, J. W., Zamora, P. G. P. and Pasquini, C., *Analytica Chimica Acta*, **285** (1994), 287.
18. Raimundo Jr., I. M. (PhD thesis, UNICAMP, Campinas, Brazil, 1995).
19. Pasquini, C. and Raimundo Jr., I. M., *Química Nova*, **7** (1984), 24.
20. Cunha, I. B. S. and Pasquini, C., *Analyst*, **117** (1992), 905.
21. Raimundo Jr., I. M., *Laboratory Microcomputer*, **13** (1994), 55.
22. Malcome-Lawes, D. J., *Laboratory Microcomputer*, **6** (1987), 16.
23. Malcome-Lawes, D. J., *Laboratory Microcomputer*, **6** (1987), 122.
24. Nomenclature, symbols, units and their usage in spectrochemical analysis. II. Data interpretation, *Spectrochimica Acta*, **33B** (1978), 241.

